# Prediabetes and cardiovascular complications study (PACCS): international collaboration 4 years’ summary and future direction

**DOI:** 10.1186/s13104-017-3017-7

**Published:** 2017-12-11

**Authors:** E. U. Nwose, R. S. Richards, P. T. Bwititi, E. O. Igumbor, E. J. Oshionwu, K. Okolie, I. C. Onyia, A. Pokhrel, P. Gyawali, J. N. Okuzor, V. M. Oguoma, F. W. Gardiner, L. Wang

**Affiliations:** 1 School of Community Health, Faculty of Sciences, Charles Sturt University, Orange Campus, Leeds Parade, Orange, NSW Australia; 2 Public & Community Health Department, Novena University, Ogume, Kwale, Nigeria; 3 Global Medical Research & Development Organization, Catholic Hospital Abbi, Albury, NSW Australia; 4 School of Biomedical Sciences, Faculty of Sciences, Charles Sturt University, Wagga Wagga, NSW Australia; 5 California Department of State Hospital, Stockton, CA 95215 USA; 6 Onyx Hospital & Maternity, Lagos, Nigeria; 7 Nepal Medical College & Teaching Hospital, Kathmandu, Nepal; 8 University of Montreal, Montreal, Canada; 9 Laboratory Department, Texas Health Resources (HMH-HEB), Bethesda, TX 76022 USA; 10 School of Psychological & Clinical Sciences, Charles Darwin University, Wagga Wagga, NSW Australia; 11 Calvary Public Hospital, Bruce, ACT Australia

**Keywords:** Cardiovascular complications, Diabetes mellitus, Early identification and intervention, Holistic healthcare management, Low-mid income countries, Prediabetes, Public health

## Abstract

**Objective:**

The prediabetes and cardiovascular complications studies proposes to develop a screening protocol for diabetes cardiovascular risk, and strategies for holistic management amongst others. Over 500 participants were recruited in the first 2 years of rural community research screening. Specific for this report, various published findings were reviewed. The objective is to summarize research outcomes and itemize limitations as they constitute basis of future directions.

**Results:**

Affordability and availability are major confounding behavioural change wheel factors in the rural community. 4.9% prevalence of prediabetes, which may be lower or non-significantly different in urban areas. Hyperglycaemia co-morbidity with dyslipidaemia (5.0%), obesity (3.1%) and hypertension (1.8%) were observed. Limitation of the study includes participants being mostly over 60 years old, which has created impetus for the Global Alliance on Chronic Diseases agenda on vulnerability of older adults to diabetes being a new direction of the collaboration. Other directions in Australia and Nepal focus on patients with chronic kidney disease with or without cardiovascular complications. This report highlights the need to translational research.

**Electronic supplementary material:**

The online version of this article (10.1186/s13104-017-3017-7) contains supplementary material, which is available to authorized users.

## Introduction

### The original research proposal

**Fig. 1:**
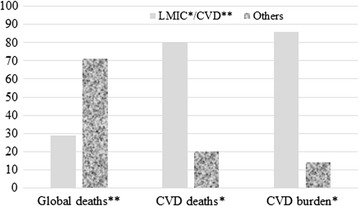
Estimated global burden and deaths due to CVD. *Deaths due to CVD vs. other diseases [4]; **LMIC vs. developed countries [5, 6]

Cardiovascular risk assessment in prediabetes and undiagnosed diabetes mellitus study has been identified as necessary, especially for low-mid income communities [1]. To substantiate the discussion of prediabetes and cardiovascular disease (CVD), a systematic review had been performed that provided insight, for instance, the need to study rural communities where lifestyles are different from the urban areas [2]. Suffice to say that some of the burdens or deaths associated with CVD are preventable since they are due to avoidable risk factors such as unhealthy diet, physical inactivity and smoking [3]. In particular, the global burdens of CVDs is rife (Fig. 1) [4–6]. As published [1], the aims of the ‘prediabetes and cardiovascular complications studies (PACCS) collaboration initiative are:

1. Diagnosis, management and monitoring of diabetes mellitus (DM) pathogenesis by laboratory methods.
2. Early and holistic intervention in DM pathogenesis in the perspective of health education as well as lifestyle changes.
3. Support system for allied healthcare professionals’ integration in the prediabetes care team.

### The proposed research design

The study is designed to be in three phases. The first phase is to identify prediabetes and undiagnosed diabetes through population screening exercise. The second and third phases will be longitudinal studies involving participants identified in the first phase as having prediabetes without dyslipidaemia, or clinically established cardiovascular disease. The second phase shall focus on preventive management including evaluation of the use of exercise, and nutrition. The third phase will include probing the development of diabetes and CVD with a view to develop a model chart for the assessment of CVD risk in prediabetes [1].

In terms of significance of the research agenda, suffice to highlight with the quote that

“Diabetes is a main ticking time bomb out there in low- and middle-income countries … that don’t have the health-care infrastructure … need to start to tackle these problems now”

[7]. That is, given the lack of data on the burden of disease, especially among the unsearched rural communities of low-mid income countries (LMIC) means lack of evidence for policy makers to make the health services unavailable, a research program focusing on this issue is imperative. For this reason, the PACCS agenda has been designed to occur in phases as ongoing since 2013.

### The objective of this report

The purpose of this report is to present a summary of our research outcomes since the beginning in 2013, including how the limitations encountered so far have formed basis for future directions.

## Main text

### Methods

Volunteers recruited included 74 collected in 2013 [3, 8], and 422 collected in 2014 [9, 10]; which have been analysed and reported. Another volunteers of 2015 and 2016 have been screened and still being analysed. Body mass index (BMI), blood pressure and waist circumference, blood glucose level, and lipid profile were measured. The *Stanford Patient Education Research Centre* questionnaire was used to collect data on knowledge, attitude and practice (KAP) based on established formats [3, 8]; while and World Health Organisation (WHO) Global Physical Activity questionnaire was used to access physical activity domains amongst others.

In all cases of data collection, purposive recruitment of participants were by invitation for public health lectures in health facilities and at schools, after which, inclusion of volunteers was based on consent to participate and being ≥ 18 years old. Clinical assessments and laboratory tests were carried out according to standard operational procedures in the health facilities. Different inferential statistics were adopted in the various pieces of studies. For instance:

KAP analysis on ‘questions about lifestyle, occupational backgrounds and visiting healthcare facilities’ involved “determining percentage of participants whose health status require healthcare”, by stratifying participants’ opinion on their general health condition, and then cross-checking with responses on the questionnaire regarding visiting clinic and/or monitoring for CVD risk factors they have performed or have been done on them” [3]. Preliminary study on metabolic syndrome and prediabetes also involved determination of percentage prevalence based on (and comparison of) commonest three criteria—Third Adult Treatment Panel, International Diabetes Federation, and WHO [8]. Prevalence of cardiovascular disease risk factors among a Nigerian adult population: relationship with income level and accessibility employed WHO STEP wise questionnaire to generate socioeconomic data of the participants, amongst others. “Multivariate analyses were performed to assess any difference between the geographical locations and SES indicators, and prevalence of CVD risk factors and access to CVD risk screening” [9].

In the association of physical activity with metabolic syndrome, ‘World Health Organisation (WHO) Global Physical Activity Questionnaire’ was adopted. Inferential statistics involving “Cross-tabulation between dichotomous variables of total physical activity and metabolic syndrome risk factors, education, work status and income status were generated to assess the prevalence of physical activity/inactivity across the different socio-demographic/economic variables and metabolic syndrome risk factors” [10]. In the evaluation of Cardiovascular disease risk factors in a Nigerian population with impaired fasting blood glucose level and diabetes mellitus, optimal discriminant analysis and ‘Hierarchical Optimal Classification Tree Analysis’ was employed [11]. More details on these research methods including statistics analyses were as published [3, 8–11].

### Results: synopsis of 4 years research outcome

The following numbered points are some of the results summaries of various research outcomes since the last 4 years. The first three are on ‘screening and evaluation of prevalence—phase 1 of study’, while the forth is in view of phase 2 of the research program. For the purpose of a ‘summary update’, these results are from several studies has provided with reference to where each report has been published.

1. Prediabetes: The preliminary observation of prediabetes was 6% in females compared to 3% in males, but the overall prevalence of metabolic syndrome (MS) was less in females than males [8]. Further data collection showed no statistical difference between gender groups [9]. There is estimated 6.4% prevalence of prediabetes in the rural community [9], which may be higher or non-significantly different in urban areas (Fig. 2). Collaborative consolidated data indicate overall prevalence of 5.8% impaired fasting glucose and 3.1% diabetes [11].

**Fig. 2:**
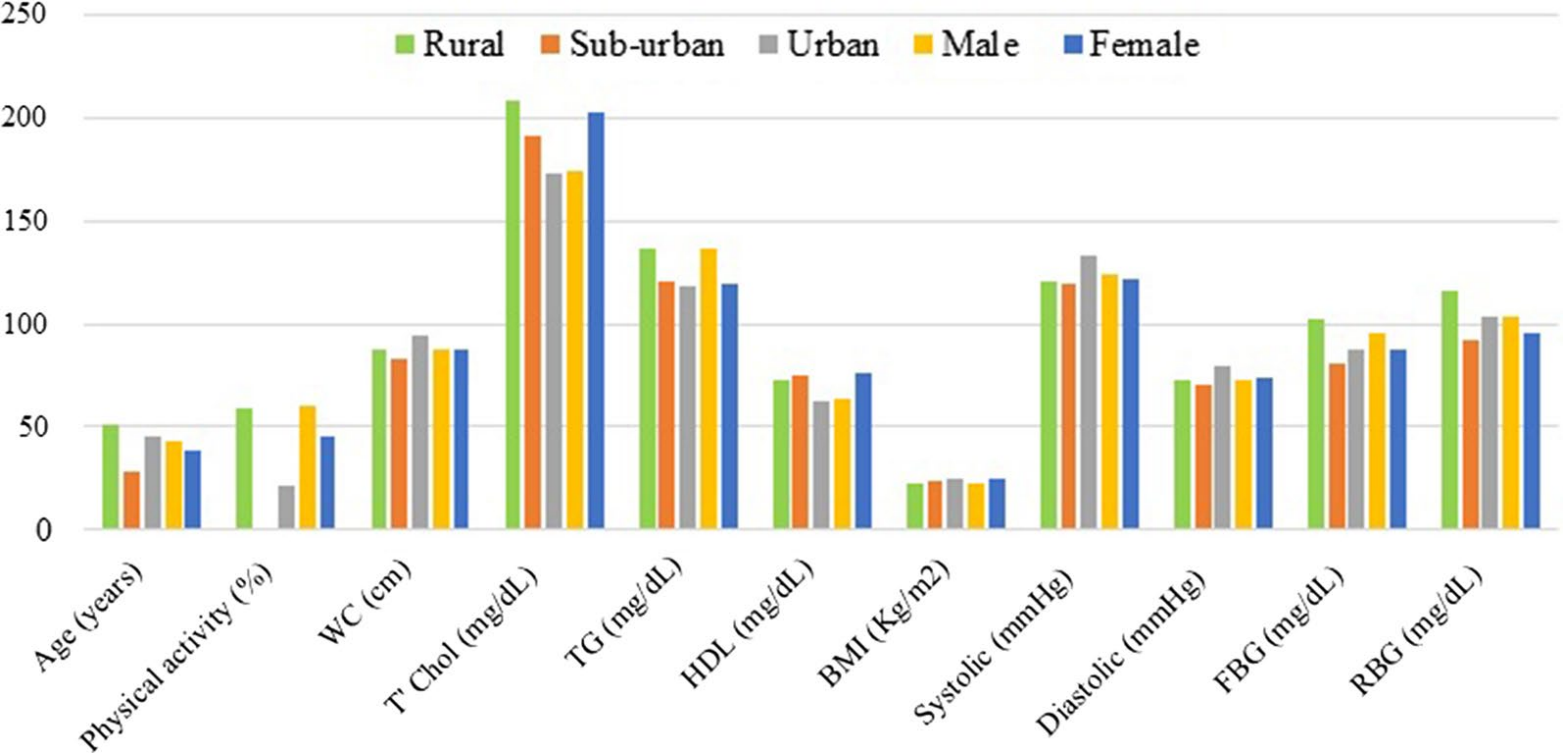
Comparisons of average levels anthropometric and biochemical parameters between gender group and ‘rural vs. urban clusters’
2. Prevalence of hypertriglyceridaemia and hypo-HDL observed in 2013 compared to 2014 were not exactly the same. Here, we report that prevalence of abnormal HDL-cholesterol, total cholesterol and triglyceride averaged 22, 45 and 37%, respectively (Additional file 1) [8, 9]. The gender differences in prevalence are reflected in average levels in Fig. 2.
3. Percentage of population with and without prior assessment vs. prevalence: first, over 70% of the participants in the rural community are yet to have health check or screening for DM and CVD risk [3]. This indicates proportion of participants in the population without priori assessment of MS. Second, in terms of CVD risk factors associated with IFG, co-morbidity with dyslipidaemia (5.0%) was the highest followed by obesity (3.1%) and hypertension (1.8%) [11]. Relative to the first point, this indicates prediabetic participants with MS (Table 1).

**Table 1:** Prevalence (%) of participants with prior assessment vs. DM and MS factors

	Yes	No	Unsure
Prior assessment^a^			
Blood sugar level	13.5	74.3	12.2
Lipid profile	8.1	78.4	13.5
Overweight	1.4	85.1	13.5
Prevalence^b^			
Prediabetes only	5.8	94.2	Not applicable
Diabetes only	3.1	96.9	
Diabetes + dyslipidaemia	2.6	97.4	
Diabetes + hypertension	1.9	98.1	
Diabetes + obesity	2	98	
Prediabetes + dyslipidaemia	4.8	95.2	
Prediabetes + hypertension	2.6	97.4	
Prediabetes + obesity	3.1	96.9	

^a^Participants with or without prior assessment [3]

^b^Prevalence rates based on research data [11]
4. Physical activities: analysis indicates that women are significantly less physically active than men (Fig. 2) [3, 10].

The results in Table 1 show, for instance, that only 13.5% have assessed their diabetic status while there is up to 5.8 prevalence of prediabetes that could benefit from early intervention. Or in other words, that 78% participants are yet to assess their dyslipidaemia status whereas among those who assessed, there is 2.6 and 4.8% comorbidity with diabetes and prediabetes, respectively.

### Discussion

A major finding being reported in this summary is that the prevalence of prediabetes in the rural community is much higher than speculated. We have reported on CVD risk assessment in prediabetes and undiagnosed diabetes mellitus (UDM) in Nigeria [1]. Appropriate glycaemic control is crucial to delay or prevent the progression of diabetes [12], and a few points are pertinent to emphasize, which made this report update imperative.

1. Both physical inactivity and prediabetes appear higher in the women by comparison with men in the study area (Fig. 2), and such observations can serve as basis for gender specific diabetes education and exercise regimen.
2. Higher prevalence of diabetes and prediabetes in the rural community (Fig. 2); disagrees with data of the Diabetes Association of Nigeria that indicates a < 1% prevalence in a particular rural community and 11% in urban Lagos [13], but agrees with another report from the United States that the disease may be more prevalent in rural communities [14]. Perhaps, in future discourse and research on rural–urban disparities, recourse should be made to the genetic make-up of subpopulations compared. For instance, in our study, the urban subpopulation comprised of individuals of the same Ukwani ethnicity as the rural community.
3. Prevalence of dyslipidaemia and/or obesity (Fig. 2; Table 1) indicates that relatively equal number (< 5%) of the people in the Nigeria require only lipid or BMI model of cardiovascular screening, while over 95% may benefit from either of the two models. This is relevant for service delivery, especially as lipid profile testing is mostly unaffordable.

In addition to diabetes whose incidence is on the rise, there is the issue of oxidative stress, which is a CVD risk factor that is common in LMIC such as Nepal and Nigeria [15–18]. There is scientific underpinning for exercise therapy and nutrition to manage and prevent oxidative stress in diabetes [19–21] thus CVDs. Given our observation on physical activities, lifestyle changes for stress management and the behavioural change wheel remains one of our research in phase two.

CVD is established as a leading contributor to the burden of diseases and mortality [6, 22], and estimated figures are presented (Fig. 1). Some of the burdens or deaths are preventable since they are due to avoidable risk factors such as unhealthy diet, physical inactivity and smoking [3]. Yet, there is paucity of reports on DM and dyslipidaemia, and where the reports are available they are based on studies in urban areas [2]. Thus, it is important to equally focus research in urban and rural areas.

In conclusion, this study seeks how best to tackle diabetes problem in rural communities of LMIC. This summary emphasizes the prevalence of diabetes and prediabetes in LMIC being higher than speculated. The report highlights the need for translational research in rural communities to enhance management of DM and its CVD complications; as well as indicates the prospect of international collaborations.

## Limitations

Every research sampling method has merits and demerit. In this occasion, the purposive, but convenience recruitment method creates its own bias such as sample size. Consciousness of such bias necessitated the invitation of other researchers for synergistic consolidated data analysis [23]. The three limitations that constitute basis for future research directions are worthy of mention here, and will be elaborated in supplementary document (Additional file 2).

Firstly, there is albeit better-than-expected good health of the Nigerian rural population viz: the prevalence of prediabetes observed is less than hypothesized. The implication is that recruitment into phase 2 and 3 of original proposal is slow. These limitations have led to the future direction involving a proposal of the consortium in Nepal whose broad objective is assessment of CVD complications in CKD patients undergoing haemodialysis through routinely measured laboratory parameters.

Secondly, in the original proposal, 3 centres were identified for data collection. Lack of funding as well as logistics resulted in data collection only occurring in the Nigerian centre. However data collection has now started in Australia, as was the intention. The proposal was that data can be extracted from the electronic medical records to determine. The future direction is whether the targets on blood pressure and/or glycaemic control in patients with CKD and diabetes are met, respectively; as well as if blood viscosity changes correlates with glycated haemoglobin levels.

Thirdly, in the Nigerian centre where screening is occurring, consent and participation has been mostly by the older adults. This limitation has provided impetus to develop proposal for studying increased vulnerability of older adults to DM and its CVD complications [24], in line with Global Alliance on Chronic Diseases agenda. An expatiation of these three future research directions are presented in supplementary document (Additional file 2—future directions’).

## Additional files

**Additional file 1.** 2 years’ (2013 and 2014) data on observed prevalence* of lipidaemia. The observation of prevalence of dyslipidaemia in 2014 was apparently different from the preliminary data of 2013. Given the study is the same community, but different cohort of volunteer participant, this table presents the averages for the 2 years data.

**Additional file 2.** Revision of proposal—three future research directions. This article is founded on the premise of ongoing research activities whereby there are additional research directed, which could not be contained within the words limit of this journal policy. Additional file 2 is a ‘supplementary’ provision for the extra information, instead of publishing it as a 2nd (albeit short) paper in another journal.

## Abbreviations

BMI: body mass index
CKD: chronic kidney diseases
CVD: cardiovascular disease
DM: diabetes mellitus
HDL: high density lipoprotein cholesterol
KAP: knowledge, attitude and practice
LMIC: low-mid income countries
MS: metabolic syndrome
PACCS: prediabetes and cardiovascular complications studies
WHO: World Health Organization
