# Supplementary material for: Prediabetes and cardiovascular complications study (PACCS): international collaboration 4 years’ summary and future direction
Source: BMC Res Notes. 2017 Dec 11;10:730. doi: 10.1186/s13104-017-3017-7 (PMC5725921; doi:10.1186/s13104-017-3017-7)
Supplement: Supplementary file 1 — Additional file 1. 2 years’ (2013 and 2014) data on observed prevalence* of lipidaemia. The observation of prevalence of dyslipidaemia in 2014 was apparently different from the preliminary data of 2013. Given the study is the same community, but different cohort of volunteer participant, this table presents the averages for the 2 years data. [file 13104_2017_3017_MOESM1_ESM.docx]

Additional file 1. 2years’ (2013 and 2014) data on observed prevalence* of daemia

| **Lipids** | **Year** | **All** | **Females** | **Males** |
| --- | --- | --- | --- | --- |
| High TC | 2013 | 17/71 (23.9) | 11/35 (31.4) | 6/36 (16.7) |
|  | 2014 | 150/382 (29.3) | 102/240 (42.5) | 48/142 (33.8) |
|  | 2years combined | 167/453 (36.9) | 113/275 (41.1) | 54/178 (30.3) |
| High TG | 2013 | 5/71 (7.0) | 3/35 (8.6) | 2/36 (5.6) |
|  | 2014 | 93/382 (24.3) | 47/240 (19.6) | 46/142 (32.4) |
|  | 2years combined | 98/453 (21.6) | 50/275 (18.2) | 48/178 (27.0) |
| Low HDL | 2013 | 32/71 (45.1) | 12/35 (34.3) | 20 (55.6) |
|  | 2014 | 69/382 (18.0) | 37/240 (15.4) | 32/143 (22.4) |
|  | 2years combined | 101/454 (22.2) | 49/275 (17.8) | 52/179 (29.1) |

*Percentage prevalence in brackets
