# Supplementary material for: Prediabetes and cardiovascular complications study (PACCS): international collaboration 4 years’ summary and future direction
Source: BMC Res Notes. 2017 Dec 11;10:730. doi: 10.1186/s13104-017-3017-7 (PMC5725921; doi:10.1186/s13104-017-3017-7)
Supplement: Supplementary file 2 — Additional file 2. Revision of proposal—three future research directions. This article is founded on the premise of ongoing research activities whereby there are additional research directed, which could not be contained within the words limit of this journal policy. Additional file 2 is a ‘supplementary’ provision for the extra information, instead of publishing it as a 2nd (albeit short) paper in another journal. [file 13104_2017_3017_MOESM2_ESM.docx]

Additional file 2

**Revision of proposal – three future research directions**

1. ***Revision of proposal with additional centres in Nepal***

The research program and proposal are now being revised to include a screening and data collection centre in Nepal, as well as a co-researcher specifically at the Medical College & Teaching Hospital, Kathmandu. This centre has potential to run large scale epidemiological studies, whereby sufficient participants may be recruited for phase 2. The proposal of the consortium in Nepal is on chronic kidney diseases (CKD) being a global epidemic with 10.2% prevalence reported in Nepal [1], and estimation that patients with reduced kidney function are more likely to die of CVD than to develop end stage renal disease [2]. Various pathophysiological pathways and explanations have been suggested to explain the mechanisms of CVD complications in CKD and these include uraemia-related risk factors, such as anaemia and alterations in calcium/phosphorus metabolism, that also play a role in promoting CVD [3].

Moreover, whole blood viscosity (WBV) has been shown to be an independent risk factor of CVD [4], and in the Edinburgh Artery Study (a prospective study of a random population sample), WBV was shown to be associated with cardiovascular complications (ischemic heart disease and stroke). The relative risk of cardiovascular events due to elevated WBV was as strong as established risk factors, such as increased cholesterol and high blood pressure. Thus, the assessment of WBV in CKD can provide information on CVD risk in CKD patients. Since, CVD complications are common in CKD patients, therefore assessment of CVD risk in this cohort can help in early identification of the patients at CVD risk. The study aims to evaluate the risk of cardiovascular complications in CKD patients not undergoing haemodialysis using common laboratory parameters assessable to developing communities.

While broad objective of the consortium is assessment of CVD complications in CKD patients undergoing haemodialysis through routinely measured laboratory parameters, the specific objectives include:

1. Measurement of the levels of calcium and phosphorous in CKD patients and assess CVD risk. This is considering the knowledge that Calcium and phosphate dis-metabolism is a feature of CKD and this may lead to vascular calcification that is a risk factor of CVDs [5].
2. Assessment of WBV e.g. from routinely measured laboratory parameters (total protein and haematocrit), in CKD patients and evaluating WBV as a risk for CVD.
3. Measurement of traditional CVD risk factors viz: lipid profile in CKD patients and evaluating its association with calcium-phosphorous product and WBV.
4. ***Ongoing data collection and analysis in Australia***

In line but prior to the proposal from Nepal, it was acknowledged in research discussion that there are large numbers of patients with CKD, diabetes and hypertension (HT) in clinical practice. The proposal was that data can be extracted from the electronic medical records (EMR) to determine whether the targets on blood pressure (BP) control in patients with CKD and diabetes are met, and the potential barriers. The same thoughts apply to whether blood glucose control targets are met in DM patients, and if blood viscosity changes correlates with glycated haemoglobin (HbA1c) levels. Data are now being collected from EMR and analysed in this regard.

Preliminary findings from evaluations of (1) *diabetes services* and (2) *cardiac rehabilitation program* indicate that the cardiac rehabilitation program is effectively reducing risk factors among DM and hypertension patients, thus leading to reduced risk of future cardiovascular and renal disease.

1. ***Global alliance on chronic diseases (GACD) agenda***

This research proposal has two main hypotheses – (a) lifestyle changes required to manage DM and MS are the same as for populations living in rural and urban cities; and (b) regardless of absence of ‘big name’ fast foods in rural communities, junk foods and unhealthy lifestyle contributing to DM and MS requiring intervention is the same in the rural communities as in the urban areas. Interventional changes in cultural and psychosocial lifestyle benefit management of DM in the primary healthcare, especially among vulnerable older adults.

***Summary of preliminary observation***: The vulnerability of older adult to diabetes and cardiovascular complications has underpinned the GACD agenda [6]. Given that lifestyle’s impact on this vulnerability is still to be clearly established, the preliminary study sought to evaluates lifestyle scores in different subgroupings of age and health status. The objective was to determine any difference between age or disease subgroups in lifestyle scores.

203 participants (female//male = 141//62) ranging from 18 – 90 years old underwent anthropometric measurements at Catholic Hospital, Abbi, Nigeria. Vital signs and BMI parameters were collected according to clinical protocols at the hospital. Lifestyle scores were determined from 12 questions including alcohol consumption, cigarette smoking, daily routines, health status, and physical activities. Data analysis included determination of average age in different health conditions, percentage impacted by ill-health, lifestyle scores in age-groups, and regression for impact of age. Average age of healthy subpopulation is 39 years while diabetes, hypertension and obesity subpopulations are 58 years, 64 years and 56 years, respectively. Percentage of age-groups whose daily activities are unaffected by ill-health decreases with age. However, the percentage or proportion of the age groups that constitute disease sub-populations is significantly different, but distribution of age-groups into health conditions shows no statistical difference.

Hence, this new direction has a focus on lifestyle changes required to manage DM and dietary habits in rural communities. As part of the ongoing future research direction, preliminary result shows that walking is the commonest form of exercise and significantly decreases with age. Yet, interference on daily activities also increases with age, and may impact on vulnerability of older adults to diabetes.

**References**

1. Tsukamoto Y, Wang H, Becker G, Chen HC, Han DS, Harris D, Imai E, Jha V, Li PK, Lee EJ *et al*: Report of the Asian Forum of Chronic Kidney Disease Initiative (AFCKDI) 2007. "Current status and perspective of CKD in Asia": diversity and specificity among Asian countries. *Clinical and experimental nephrology* 2009, 13(3):249-256.

2. Collins AJ, Kasiske B, Herzog C, Chavers B, Foley R, Gilbertson D, Grimm R, Liu J, Louis T, Manning W *et al*: Excerpts from the United States Renal Data System 2004 annual data report: atlas of end-stage renal disease in the United States. *Am J Kidney Dis* 2005, 45(1 Suppl 1):A5-7, s1-280.

3. Regmi P, Malla B, Gyawali P, Sigdel M, Shrestha R, Shah DS, Khanal MP: Product of serum calcium and phosphorus (Ca x PO4) as predictor of cardiovascular disease risk in predialysis patients. *Clin Biochem* 2014, 47(1-2):77-81.

4. Gyawali P, Richards RS, Tinley P, Nwose EU: Hemorheology, ankle brachial pressure index (ABPI) and toe brachial pressure index (TBPI) in metabolic syndrome. *Microvasc Res* 2014, 95:31-36.

5. Palit S, Kendrick J: Vascular calcification in chronic kidney disease: role of disordered mineral metabolism. *Current pharmaceutical design* 2014, 20(37):5829-5833.

6. Frood A: Funding: Global Alliance for Chronic Diseases tackles diabetes. *The Lancet* 2013, 382(9897):1014.
